# Analgesic Techniques in Hip and Knee Arthroplasty: From the Daily Practice to Evidence-Based Medicine

**DOI:** 10.1155/2014/569319

**Published:** 2014-11-17

**Authors:** Denisa Madalina Anastase, Simona Cionac Florescu, Ana Maria Munteanu, Traian Ursu, Cristian Ioan Stoica

**Affiliations:** ^1^Department of Anesthesiology, Clinical Hospital of Orthopedy Foisor, Bulevardul Ferdinand, No. 35-37, 021382 Bucharest, Romania; ^2^Department of Orthopedics, Foisor Clinical Orthopedics Hospital and “Carol Davila” University of Medicine and Pharmacy, 020022 Bucharest, Romania

## Abstract

Total hip arthroplasty (THA) and total knee arthroplasty (TKA) are major orthopedic surgery models, addressing mainly ageing populations with multiple comorbidities and treatments, ASA II–IV, which may complicate the perioperative period. Therefore effective management of postoperative pain should allow rapid mobilization of the patient with shortening of hospitalization and social reintegration. In our review we propose an evaluation of the main analgesics models used today in the postoperative period. Their comparative analysis shows the benefits and side effects of each of these methods and guides us to how to use evidence-based medicine in our daily practice.

## 1. Background

Primary total arthroplasty of the hip or knee is a common surgery today with an increasing incidence related to age. The mean age at surgery for TKA is 70 years, but there is a tendency worldwide to increase the proportion of younger patients (the age interval between 55 and 64 years) [1–3]. To allow comparison between data from different arthroplasty national registers, the term “age-standardised” was introduced as a statistically corrected result for age structure of the population [4] and the “rate” as the number of the knee TKA per 100.000 inhabitants is used. The leaders are USA (221,5) and Austria (186,3), Switzerland (173,6) [5], Germany (132,5) [6]. TKA data from Registers Nordic Association (including the 4 countries Sweden, Norway, Denmark, and Finland) show 151 814 knee prostheses till 2011 [7] with a higher incidence in Denmark (123) in 2007 than in Sweden (115) and Norway (75) [1].

Besides increasing the number and the age of patients receiving prosthetic joint, a number of comorbidities are associated with increasing age. According to a national cohort report from 2009, 32,6% of the patients with TKA had three comorbidities or more and the most common ones are hypertension (67,8%), diabetes (20%), and obesity (19,8%) [8]. Therefore, there was an almost parallel evolution of surgical techniques and methods of analgesia, allowing effective pain control, rapid mobilization of patients with reduced side effects and no damage to existing comorbidities.

Effective treatment of postoperative pain continues to be a challenge because it influences the surgical outcome [9] and for prosthetic joints pain management is a must for early mobilization and functionality of the new joints.

Relationship between analgesic technique and the immediate and remote postoperative outcome and success of surgery is not new, and postoperative pain assessment using visual analogue scale (VAS) and opioid requirements is the primary outcome variable in most studies.

That is why, since 1996, the pain was declared the fifth vital sign by the American Pain Society [10] (http://www.americanpainsociety.org/uploads/pdfs/npc/section_2.pdf).

Moreover, effective treatment of postoperative pain was the most important factor for early mobilization, shortened hospital stay, and discharge [11].

## 2. Comparative Analgesia Techniques

Epidural analgesia offers advantages over systemic opioid administration by patient controlled analgesia (PCA) and has been the gold-standard for a long time in postoperative pain control in THA.

Regarding TKA, Mahoney et al. administered bupivacaine and morphine continuously on epidural catheter and reported good-to-excellent analgesia but with a high incidence of adverse effects related to epidural catheter and opioids [12]. The necessity for other solutions with a lower incidence of opioid-associated side effects was obvious.

Moiniche et al. study the effect of pain relief with balanced analgesia on postoperative convalescence parameters in 20 patients scheduled for TKA [13]. At 48 hours the epidural analgesia group had significantly lower pain scores, but no important differences were observed between groups related to ambulation, daily patient activity, or hospital stay [13].

Opioid dose reduction is one of the important goals of postoperative analgesia, to reduce both side effects (nausea, itching, vomiting, respiratory depression, and ileus) and consecutive sedation, leading to a delay in patient mobilization. In addition to careful monitoring, symptomatic management is required for each type of secondary reaction that occurred, leading to a higher consumption of drugs and therefore higher costs of hospitalization.

Understanding the mechanisms involved in nociceptive acute pain, identifying the effect of opioid-induced hyperalgesia and persistent postsurgical pain by regional anesthesia, directs the development of new drugs or different analgesia regimens for improving postprosthetic outcome [14].

Therefore, epidural technique has quickly gained popularity compared to intravenous opioid analgesia. Several studies have shown that epidural analgesia may reduce the risk of myocardial ischemia and tachyarrhythmia in coronary high-risk patients proposed for elective noncardiac surgery [15] and even to those proposed for cardiac surgery [16], as well as in major abdominal and thoracic surgery [17].

Besides the protective effect in high-risk cardiac patient, epidural analgesia has a beneficial effect on lung function with decreased incidence of postoperative pulmonary morbidity as shown by meta-analysis of Ballantyne et al. [18]. All these positive effects may become important in the elderly patient with comorbidities.

Cochrane data of evidence-based medicine led a study in 2003, trying to answer the question, “Is the lumbar epidural analgesia more efficient than the systemic analgesia or spinal analgesia for pain relief after elective primary hip or knee arthroplasty?” [19]. Their conclusion was unequivocal: there is not enough evidence to draw conclusions about the efficiency of epidural analgesia compared with systemic analgesia, epidural analgesia effect on postoperative serious complications, the functional outcome, or length of hospital stay. The benefit of epidural analgesia on pain is limited to the first 4–6 postoperative hours and epidural administration of local anesthetic or a local anesthetic and opioid combination may be more effective than the epidural opioid administration only.

In the same year, a study compared the analgesic effectiveness and side effects of intrathecal morphine sulphate in small doses, between 0.0 and 0.3 mg, on 40 patients with THA and 40 patients with TKA [20]. The conclusion was consistent with other studies: analgesic needs are higher in total knee replacement than in THA where the pain is moderate [21], and the combination of low-dose intrathecal morphine 0.2 mg with standard dose of morphine administered on the PCA provides good-to-excellent analgesia in most patients with THA but not in those with TKA, thereby reducing the consumption of morphine administered systemically.

Peripheral nerves blocks (PNB) offer advantages over epidural analgesia for the TKA patients. Ganapathy as well as Capdevilla used a modified 3-in-1 Winnie continuous femoral block [22, 23] with local anesthetic, compared to continuous epidural. The results were a lower quantity of opioid required to control pain at rest and during passive mobilization and a better articular mobility in the immediate postoperative period [22]. The epidural group had significantly elevated incidence of urinary retention, dysesthesia, and arterial hypotension [23]. Singelyn et al. showed 4 times less incidence of secondary effects with a femoral catheter compared to an epidural one [24] and Chelly et al. showed more cardiovascular stability and less nausea and vomiting [25].

Some of these better results may be due to the new mixture used by Capdevilla with the femoral catheter: lidocaine 1% and clonidine plus morphine. The usefulness of these adjuvants in brachial plexus block had been demonstrated by Singelyn et al. [26] and Bernard and Macaire [27].

Continuous administration using a femoral catheter is more useful than single shot but with minimal impact on hospital length of stay and long-term functional recovery [28].

Among local anesthetics, lidocaine 1% performs a better motor block than bupivacaine 0.25% and seems to be more efficient in alleviating quadriceps muscle spasm [29]; this spasm is the main effect of postoperative TKA pain that impairs early articular mobilization; at the same time a good quality femoral block may be associated with weakness of the muscle and risk of falling during the postoperative recuperation [30].

Fowler's meta-analysis from 2008 undertook a systematic review of all randomized trials comparing epidural analgesia with peripheral PNB for TKA. There was an evaluation on 464 patients of morphine consumption, epidural and PNB side effects, and the patient satisfaction [31]. The conclusions are as follows: (1) there was no significant difference in pain VAS scores between epidural and PNB for the first 24 hours; (2) patient satisfaction was higher with PNB in only two studies [32, 33]; (3) the addition of sciatic nerve block improves the quality of analgesia by reducing posterior knee and calf pain, corresponding to the area innervated by the sciatic nerve [34–38]. Fowler's analysis failed to demonstrate inferior analgesia for patients without sciatic block between 0 and 24 h after operation; (4) neurological complications referred to sphincteric disturbance in approximately 250 patients who received an epidural analgesia whereas no neurological complications were reported in the 766 patients from the PNB group. Hypotension associated with epidural analgesia occurred more frequently among the peridural patients and may contribute to end organ ischaemia or infarction if left untreated. This is the reason why epidural analgesia is not appropriate in the ward setting in every institution. If general anaesthesia is not indicated, spinal anesthesia combined with PNB provides a good analgesia with a lower incidence of neurological complications than epidural analgesia in this risk patients group (older age, degenerative spinal disorders, and chronic anticoagulation) [31, 39, 40].

Advantageous for femoral nerve block (FNB) is pain reduction related to significant reduction of quadriceps spasm, secondary after TKA and therefore it improves toleration of passive motion [41]. The FNB critics reproach quadriceps weakness on the operative side compared to the controlateral side, but in the Fowler's analysis this is unclear [31].

Ilfeld et al.'s study in 2010 introduces the concept of continuous ambulatory FNB for 4 days after a TKA discharge [42]. Beaupre et al.'s study brings back in 2012 the idea of a preemptive multimodal analgesia plus FNB protocol on rehabilitation, hospital length of stay, and postoperative analgesia after TKA [43]. In the study a combination of Oxycodone controlled release and celecoxib is used as a preemptive analgesia before the surgery. The study reflects the FNB efficacy in standard clinical conditions, although it was not randomised. There are more quadriceps motor blocks reported in the FNB group; however, no patient experienced any falls, and there were no delays in rehabilitation program secondary to FNB and no significant differences between groups in terms of pain scores, knee flexion, or hospital stay. The only restriction to use widely FNB is the accidental fall secondary to quadriceps weakness limiting an aggressive postoperative rehabilitation [30].

Regarding the quadriceps weakness secondary to FNB, Wasserstein concluded that single-shot FNB did not increase the fall's risk. Advanced age, obesity, and continuous femoral nerve blockade are independent risk factors for inpatient falls after primary total knee arthroplasty [44].

The evidence-based data regarding FNB are as follows: continuous FNB provides better pain relief than that attained with single-injection FNB and information was insufficient for review authors to conclude on the comparison of FNB with local infiltration analgesia or oral analgesia or on the safety of the various analgesic techniques [45].

A modern and easy identification method of the femoral nerve is the ultrasound. For experienced hands, the femoral nerve block under the echographic guidance has a fast installation with a high success rate [46]. The use of ultrasound in regional anaesthesia for femoral nerve is not a new idea [47, 48].

The sciatic nerve block contribution is still controversial. Pros are Cappelleri et al. who concluded that continuous sciatic nerve block improves analgesia, decreases morphine request, and improves early rehabilitation compared with single-injection sciatic nerve block [49], in contrast with Abdallah and Brulls' review, who cannot define the effect of adding sciatic nerve block to FNB on acute pain [50].

The knee innervation is mainly covered by the femoral, sciatic, and obturatorius nerves, excepting few areas of the classical incision that are innervated by the lateral cutaneous femoral nerve. The injection of the femoral nerve catheter even on a large volume of local anesthetic or the distribution of the solution more cranially does not cover the obturatorius nerve in terms of analgesia [35, 51, 52]. So the idea arose to add the analgesia in the territory of the obturatorius nerve to the combination of the femoral/sciatic nerve blocks; 2 studies confirmed hypothesis [53, 54].

Adductor canal block is a sensory blockade that provides analgesia in the territory of obturatorius nerve and saphenous nerve. There are only two studies related to the efficiency of adductor canal block analgesia compared with other established techniques in TKA, FNB. Adductor canal block preserved quadriceps muscle strength better than FNB and therefore promotes early ambulation, without a significant difference in postoperative pain [55, 56].

The psoas block analgesia should be theoretically superior to that of the femoral nerve block in TKA. Paradoxically, Morin found that the analgesia via femoral/sciatic nerves blocks combination is superior to the psoas block with decreased need for opioid [35].

Decreasing opioid consumption can be achieved by using a combination of analgesics belonging to different pharmacological classes. The multimodal analgesia is actually a balanced analgesia through a multimodal approach to postoperative pain, with synergistic effect of various analgesics and consecutive reduction of side effects associated with them by lowering doses [33].

This synergistic analgesic effect can be achieved by the following: (1) peripheral techniques using local anesthetics, nonsteroidal anti-inflammatory agents, opioids, and other analgesics or (2) peripheral nerves blocks with the use of local anesthetics and *α*-2 adrenergic agonists or (3) subarachnoid or epidural administration of local anesthetics, opioids, and *α*-2 adrenergic agonists or (4) combinations thereof.

In a systematic review of analgesics and anesthetics interventions dedicated to optimal pain influencing primary hip arthroplasty, Fischer et al. use in their analysis the Cochrane protocol and evidence-based recommendations are the following: PROSPECT group recommended for postoperative analgesia general anesthesia combined with peripheral nerve block or intrathecal administration of local anesthetic and opioid [40]. Moreover, the primary anesthetic technique must be combined with paracetamol and nonsteroidal anti-inflammatory agent, opioid stronger or weaker depending on needs, including gabapentin [57].

There is evidence of multimodal approach to pain, suggesting that that the use of analgesics in different pharmacological classes improves postoperative pain control in patients with arthroplasty.

Nonsteroidal anti-inflammatory drugs and cyclooxygenase-2 inhibitors added systemic route have a synergistic effect with local anesthetic in most cases and a safety profile regarding side effects [31]. Regarding nonspecific nonsteroidal anti-inflammatory drugs, there is evidence of a clinically relevant local effect in peripheral administration of intra-articular administration [32].

Several studies are dedicated to the adding of gabapentin to the multimodal analgesia in arthroplasty. Mathiesen starts from the evidence that gabapentin is effective in reducing postoperative pain and opioid consumption [58]. He studied the effect of administration of pregabalin 300 mg p.o. and dexamethasone 8 mg i.v. on postoperative pain control; preoperatively patients received also paracetamol 1 g and postoperative paracetamol and morphine on PCA. The study concludes that pregabalin reduced to half postoperative morphine requirements, but this reduction was not accompanied by a decrease in the incidence of nausea and vomiting; pregabalin administration was accompanied by an increased level of sedation and in combination with dexamethasone did not provide an additional effect on the score of pain or opioid requirements [58]. Although gabapentin does not have its own analgesic action, it reduces hyperexcitability in neurons of the posterior horn caused by tissue damage.

Clarke et al. study the effect of a single dose of pregabalin 600 mg administered preoperatively or postoperatively with spinal regional anesthesia and multimodal analgesia in THA. Multimodal regimen includes preoperative administration of acetaminophen p.o., celecoxib p.o., and dexamethasone i.v. The study concludes that gabapentin added to a multimodal regimen does not reduce acute pain, opioid consumption, or chronic pain after total hip arthroplasty [59].

Over the last decade, technological progress of the types of prostheses and minimally invasive surgical approaches have resulted in a faster recovery, decreased length of stay and lower costs. Parallel analgesic techniques migrate from the central (epidural or spinal analgesia) to periphery (nerve blocks and local intra-articular injection-LIA).

Soon, the concept of intra-articular local infiltration of the wound developed by Kerr and Kohan is picked up by a lot of clinics and schools, especially Scandinavia, UK, and Australia. Kerr and Kohan have developed the type of technique “local infiltration analgesia (LIA)” for pain control after total knee and hip arthroplasty. This requires systematic infiltration of a mixture of ropivacaine, ketorolac, and adrenaline in the tissues surrounding the surgical wound to achieve satisfactory analgesia with minimal discomfort [60].

The technique is revolutionary because it allows virtually immediate mobilization; most of the 325 patients included in the study can walk with assistance in 5-6 hours after surgery and regain independent mobility in 13 to 22 hours of surgery. Pain score was satisfactory, between 0 and 3 on the VAS, and side effects were infrequent; 71% of the patients were directly discharged home after one night spent in the hospital. The study's conclusion was that “LIA is simple, practical, safe and effective pain control after THA/TKA.”

Study of Andersen et al. in 2007 starts from preliminary experience using intra-articular ropivacaine in the shoulder surgery by Gottschalk and Horn [61, 62] and also from a combination of morphine and intra-articular local anesthetic in total knee prosthesis by Mauerhan and Rasmunssen [63, 64]. Andersen et al. compare prospectively randomized effectiveness LIA (with a mixture of ropivacaine, ketorolac, and adrenaline) with epidural analgesia in THA and recommend this type of analgesia [65].

Thorsell et al.'s study, also a prospective randomized trial, found no clinically important effect of LIA on the catheter compared to peridural analgesia, although patients with LIA were mobilized faster and had a higher degree of satisfaction, but there was no reduction in the consumption of morphine [66].

In the same way the prospective randomized study of Specht finds no statistically significant analgesic differences between LIA administered on catheter and placebo administered into intra-articular space in THA [67].

The prospective, randomized Lunn et al.'s study on a larger number of patients, 120, compares the analgesic efficiency between LIA with large volumes, 150 mL, and placebo in the presence of a multimodal analgesia system composed of acetaminophen, celecoxib, and gabapentin preoperatively and analyzes the first variable pain to walk from a distance of 5 m 8 hours after the surgery. The study concludes that the administration of high-volume LIA does not reduce acute pain compared to placebo in the presence of a multimodal system that combines the three drugs in total hip prosthesis [68].

The administration of local anesthetic in the surgical wound is a reasonable approach to reduce nociceptive transmission from the source [69]. Development of LIA with multimodal analgesia regimen has allowed the extension fast-track prosthetic surgery and the appearance of specialized centers in this kind of surgery and recovery.

The Swedish National Arthroplasty Registry data show for THA a steady increase in the number of replacements under the LIA in fast-track mode, as well as for TKA, the percentage being 75%. None of the studies published till 2011 could reproduce the short duration of hospitalization reported by Kerr and Kohan original study, but the average time of hospitalization decreased from 6–10 days to 3–5 days [69].

Kehlet and Andersen create a review of randomized clinical trials on the basis of existing data at the level of the year 2011 related to efficiency of LIA with large volumes in the THA and TKA. At that time, there was little evidence to recommend in both intraoperative and postoperative THA technique of letting the catheter in the wound when given nonopioid oral multimodal analgesia; regarding TKA, evidence supported the intraoperative analgesic effectiveness of LIA but not the use of the catheter for postoperative analgesia [70].

Moreover, for TKA there are sufficient data to sustain that the compressive bandage may prolong analgesia after administration of high-volume LIA intraoperative [71]. As with the length of hospitalization, lower duration was made possible by administering an oral multimodal nonopioid medication with an optimization of care in fast-track methodology.

Another recent study dedicated to the effectiveness of LIA in the THA is Solovyova et al.'s study who come into contradiction with Andersen: LIA “per se” or followed by continuous administration of ropivacaine as part of multimodal analgesia with acetaminophen, celecoxib, and pregabalin does not bring additional benefit or reduction of the opioid consumption compared to placebo [72].

If THA has not demonstrated the effectiveness of LIA in managing conditions of multimodal analgesia, for TKA there are multiple studies that compare LIA with different analgesic techniques that demonstrate the superiority of LIA on evidence-based grounds.

Thus, Toftdahl et al. by comparing the intraoperative and perioperative analgesia of LIA with continuous femoral nerve block in TKA show a superiority of the LIA and early mobilization, without influencing the duration of hospitalization [73].

Essving et al. found the same positive analgesic LIA effect and a reduction of the postoperative morphine consumption, comparing the intraoperative versus postoperative administration only, with a high degree of patient satisfaction [74].

Spreng et al. compare epidural analgesia with large volumes LIA and local adjuvants combined such as ketorolac and morphine (or administered systemically). The study concludes that the LIA with local adjuvants reduces opioid consumption, allow a faster mobilization and a reduction in length of stay; ketorolac and morphine were more effective locally than systemically administered [75].

Dillon's study starts from the Kerr and Kohan technique and presents in a review several changes related to evidence-based data that they use in patients proposed for joint replacement: add dexamethasone to the initial mixture and use minimally invasive surgical approaches, give up the use of intraoperative tourniquet limiting edema and allowing reducing of pain associated with edema, and use bandage from toe to thigh also involved in improving analgesia associated with LIA [60].

Their experience shows that LIA is well tolerated by patients allowing early walking on the day of surgery for both THA and TKA with a good pain control and decreasing the duration of hospitalization [76].

Perlas retrospective study of 298 patients analyzed analgesia and recovery at 48 hours in patients with TKA and LIA versus continuous femoral nerve block or LIA plus adductor canal block. LIA was associated with improved early analgesia and walking in the first postoperative day; the adding of the adductor canal block allowed a faster gait with the increased incidence of discharge to home [77].

Fowler explores in a recent review the LIA with large volume in THA/TKA versus peripheral nerves blocks in terms of functional outcome, persistent postoperative pain, costs, and the revision rate. Despite the growing popularity and good postoperative analgesia the role of LIA in the context of a recovery program, the persistent postoperative pain, or efficiency compared with peripheral nerves blocks is not clear [78].

The most recent review is from 2014, analyzing 27 randomized controlled studies including 756 patients operated on with THA and 888 patients with TKA. Andersen and Kehlet explore the effectiveness of LIA in early treatment of postoperative pain, the analgesic efficiency of the surgical wound catheter in postoperative period, and if there is any impact on the duration of hospitalization. As shown earlier, LIA has similar analgesic effectiveness with administration of intrathecal morphine and epidural analgesia in THA. For TKA, most trials provide a reduction of pain with LIA and also a reduction in the amount of opioids compared to placebo. LIA is similar to continuous femoral nerve, intrathecal morphine analgesia, or epidural analgesia for early postoperative period, with a high risk of error due to different systemic analgesia between groups. Using the wound catheters for postoperative analgesia does not bring additional benefits; length of hospital stay is not related to analgesic effectiveness [79].

## 3. Conclusion

THA and TKA are surgical procedures for removal of pain and restoration of mobility to patients with gonarthrosis and coxarthrosis, which are associated with moderate to severe postoperative pain. If former analgesic approaches addressed first the postoperative pain, appearance of new operative techniques, like minimally invasive surgical approaches, has led to an adaptation of postoperative analgesia methods with the aim of more early mobilization, even on the day of surgery, and a shorter hospital stay with home discharge directly without the need for an intermediary rehabilitation center. Each of these new models has its adepts and the choice of one or another belongs to the anesthetic-surgical team according to the surgeon technique and has a single aim: a better prognosis for the patient.
